# Acceptability and functional benefits of a health promotion program in pediatric oncology: a mixed-methods study

**DOI:** 10.3389/fped.2026.1753530

**Published:** 2026-06-03

**Authors:** Catherine Demers, Johanne Higgins, Johanne Kerba, Isabelle Bouchard, Caroline Meloche, Daniel Curnier, Valérie Marcil, Serge Sultan, Caroline Laverdière, Daniel Sinnett, Isabelle Gélinas

**Affiliations:** 1 School of Physical and Occupational Therapy, McGill University, Montreal, QC, Canada; 2 CHU Ste-Justine Research Center, Montreal, QC, Canada; 3 Department of Occupational Therapy, Université du Québec à Trois-Rivières, Trois-Rivières, QC, Canada; 4 Centre for Interdisciplinary Research in Rehabilitation of Greater Montreal (CRIR), Montreal, QC, Canada; 5 School of Rehabilitation, Faculty of Medicine, Université de Montréal, Montreal, QC, Canada; 6 Department of Nutrition, Faculty of Medicine, Université de Montréal, Montreal, QC, Canada; 7 School of Kinesiology and Exercise Science, Faculty of Medicine, Université de Montréal, Montreal, QC, Canada; 8 Department of Psychology, Université de Montréal, Montreal, QC, Canada; 9 Department of Pediatrics, Faculty of Medicine, Université de Montréal, Montreal, QC, Canada

**Keywords:** acceptability, functional outcome, health promotion, mixed-methods, pediatric oncology, supportive care

## Abstract

**Background:**

The increasing number of childhood cancer survivors and their well-established risk of cancer-related morbidity strongly support the need for effective health promotion (HP) programs. Existing literature suggests that HP interventions may support behaviour change and reduce the severity of some adverse effects associated with cancer and its treatments. However, uncertainty remains regarding the acceptability of these interventions to families during the cancer journey and their effectiveness in improving children's functional outcomes. This study aimed to assess the acceptability of a HP program from the perspective of families affected by cancer and to explore whether participation in the program was associated with improved functional outcomes compared to standard care.

**Methods:**

A mixed-methods study was used. Acceptability was assessed through questionnaires completed by parents (*n* = 30) and a semi-structured exit interviews conducted with a subgroup of adolescents (*n* = 6) and parents (*n* = 12) who participated in the program. Preliminary effectiveness on functional outcomes was evaluated using an age-appropriate performance-based assessment [Movement ABC-2 (MABC-2) or Assessment of Motor and Process Skills (AMPS)] administered to children and adolescents (*n* = 45) in the intervention group and compared with a control group that received standard care (*n* = 29).

**Results:**

The HP program was perceived as acceptable and positively influenced participants’ experiences during the cancer journey. Families described the program as supportive, motivating, and emotionally meaningful. However, no statistically significant differences in functional outcomes were observed between the intervention and control group.

**Conclusion:**

Preliminary findings support the acceptability of implementing a HP program in a pediatric oncology clinical setting and suggest that a full-scale trial is warranted. Further research is needed to better understand how HP interventions can be optimized to improve functional outcomes among children and adolescents affected by cancer.

## 1 Introduction

Children and adolescents affected by cancer are surviving more than ever, with the 5-year survival rate exceeding 80% in high-income countries (1–3), compared to only 58% in the mid-1970s (4). However, improvement in survival rate has come at the cost of adverse, cumulative effects from cancer and its treatment, which can leave cancer survivors disabled or with a suboptimal level of function (5), defined as the physical, cognitive, and psychosocial abilities required to perform daily activities and participate in age-appropriate roles at home, school, and work. Reduced function across these domains may limit survivors' ability to perform everyday tasks and engage in expected or desired activities, ultimately restricting participation and diminishing independence (6). Furthermore, most survivors will develop a chronic health condition within 30 years of diagnosis (7). These complications may further limit activities and restrict participation in daily life experiences (8) as well as decrease their quality of life (9).

Developing and implementing health promotion (HP) initiatives that support the adoption of healthy behaviours, such as a healthy diet and frequent physical activity (PA), can be an effective way to prevent or minimize the impacts of adverse effects. Adopting and sustaining healthy behaviours is known to improve health in both the general and at-risk populations (10), particularly for chronic diseases, such as cardiovascular disease and diabetes, that are prevalent in the pediatric oncology population (11). Furthermore, benefiting from HP interventions in addition to standard care during the pediatric cancer continuum (i.e., from diagnosis to survivorship) could result in improved functional outcome.

The scientific literature on HP has examined the feasibility and effectiveness of HP interventions in pediatric oncology. Reviews of exercise or PA interventions during and after treatment for pediatric cancer show beneficial effects on muscle strength (12, 13), cardiorespiratory fitness (12, 13), functional mobility (12, 14), and fatigue (12, 15). A review of nutritional interventions for survivors of childhood cancer was unable to draw conclusion regarding their effectiveness but suggested that interventions designed to improve the nutritional intake could improve health behaviours in this population (16). A review of complex behavioural interventions (CBI) targeting multiple health behaviours in childhood cancer patients or survivors concluded that implementing CBI is feasible and that these interventions can potentially improve PA and dietary behaviours and diminish cancer effects on children's physical and psychological health (17). Studies that have included an acceptability assessment of CBI, defined as a construct that reflects the extent to which people delivering or receiving the intervention consider it to be appropriate (18), have concluded that most participants were satisfied with the program (19, 20) and found it helpful (20, 21). However, little research has explored participants’ views and experiences of HP programs during their cancer journey beyond brief post-intervention questionnaires. Furthermore, the vast majority of the studies included in these reviews have used assessments of health behaviours such as PA level or diet and/or health indicators such as body mass index, strength or quality of life to assess the effectiveness of their interventions. Thus, little is known about the potential of these interventions to improve the *functional outcome* among children affected by cancer (e.g., activities of daily living, school activities, play).

In the context of the evaluation of a HP program for children and adolescents diagnosed with cancer and their family, previous studies have examined the associations between the program and selected outcomes (22, 23), and compared PA levels and diet quality before and after the intervention while examining whether the degree of participation in the program was associated with changes in these outcomes (24). Building on this prior evaluation, the present study aimed to: (1) assess the program's acceptability and exploring views of participants regarding the program; and (2) determine its preliminary effectiveness to improve the functional outcome (i.e., motor and cognitive abilities) of children and adolescents affected by cancer, compared to standard care.

## 2 Methods and materials

### 2.1 Study design

We used a cross-sectional, convergent mixed-methods design embedded within a larger non-randomised, controlled pilot trial of a HP program in pediatric oncology. The primary aim of the broader study was to evaluate the feasibility of implementing a multidisciplinary, integrated intervention program for children and adolescents with cancer corresponding to a phase 1b of the ORBIT model (i.e., Refine phase) (25). The present study focuses specifically on program acceptability and preliminary functional outcomes. The mixed-method approach was selected to capture both quantitative indicators of acceptability and functional outcomes, alongside qualitative perspectives regarding participants' experience in the program. More specifically, a convergent mixed-methods integration approach was used to examine how qualitative findings contextualized and explained quantitative results related to acceptability and functional outcomes.

Data for the present study were collected at a single time point within the broader intervention framework, when participants reached or neared the end of treatment. Collecting data near the end of treatment allowed comparison of functional outcomes between participants who had received the program and those who had received standard care only.

### 2.2 Clinical context

This study was conducted as part of the *Valorization, Implication, Education* (*VIE) project*, a HP program pilot-tested at the Centre Hospitalier Universitaire Sainte-Justine (CHUSJ) (Montreal, Canada). The *VIE project* is a family-focused program that integrates three complementary components delivered by a multidisciplinary team: psychosocial support, nutrition counselling, and PA interventions. The overall objective of the program was to promote and sustain healthy behaviours aimed at preventing or attenuating long-term adverse health effects among children and adolescents with cancer across the continuum of care. Interventions were delivered throughout the course of treatment and early survivorship, and families were encouraged to engage with the different components according to their needs and clinical context. The program (24, 26) and its different components (27–30) are described elsewhere and a summary is provided in Table 1. Briefly, for the PA intervention, an individualized exercise program tailored to the participants' needs, abilities, health and functional status, and goals was developed by a kinesiologist. Intervention group participants underwent four assessments over a 2-year period, during which PA interventions were delivered with the duration, frequency, and intensity tailored to participants' needs, availability, and motivation. For the nutritional intervention, registered dieticians provided nutritional counselling and encouraged the adoption and refinement of nutritional goals every 2 months for a 1-year period. For the psychosocial intervention, a six-session problem-solving skills training intervention was offered to support parents over a 6- to 8-week period. Interventions were delivered in person during oncology clinic appointments or hospitalizations, or remotely via videoconference when needed or convenient for families. Participants in the control group received standard treatment only and completed the same measures as the intervention group at the end of the program. This study extends the evaluation of the *VIE project* by examining program acceptability and preliminary effectiveness on functional outcomes.

**Table 1:** *VIE project* components.

Components	Description
Psychosocial support	Six sessions (i.e., four individual sessions, offered to each parent, and two couple sessions) to support parents of children affected by cancer
	In blended families, each parent could participate in the program with their new partner and the individual sessions were also offered to single parents
	A manual for healthcare professionals (provider manual) provided specific instructions to convey the information in a standardized manner. A manual for parents included toolkits for individual and couple sessions, as well as strategies related to communication and dyadic coping.
	Program adapted from existing programs and based on cognitive behavioural and systemic theories, developed for this study
	Sessions provided either at the hospital, at home, or remotely
	Aim: to strengthen parents’ sense of control and problem-solving skills training (PSST). Individual sessions focused on PSST, as well as acquiring, developing, and maintaining simple problem-solving skills to meet the needs of families facing childhood cancer. The couple sessions aimed to enhance parents’ communication and resilience by improving their ability to manage real difficulties associated with childhood cancer together
Physical activity	Physical activity (PA) sessions, goal setting, and counselling for behavioural changes with a team of trained kinesiologists
	Assessment of patients’ physical fitness, quality of life, and level of PA at four time points (baseline, 1-year post-diagnosis, 2-year post-diagnosis, end of the study)
	Sessions conducted at the hospital during the medical appointments or hospitalization, or remotely
	Aim: to promote PA during and after the treatment as well as the adoption of an active lifestyle for the participants and their family
Nutrition	Personalized assessment, goal setting, and counseling for behavioural changes with a registered dietitian (RD)
	Initial assessment, then follow-up visits every 2 months for the first year, and as needed thereafter
	Personalized counseling focused on addressing nutritional issues related to cancer or treatments and promoting healthy eating behaviours
	Aim: to ensure normal growth and development of the patient, weight maintenance after treatment, and prevention of long-term health complications

### 2.3 Participants

We recruited participants at the oncology department of CHUSJ between February 2018 and December 2019. Parents and children newly diagnosed with cancer who were receiving treatment and met the inclusion criteria were eligible to participate in the intervention group. Inclusion criteria were as follows: (1) diagnosed before the age of 21, (2) treated with radiation therapy and/or chemotherapy, (3) within 12 weeks of diagnosis; and (4) ability to provide informed consent (provided by parents or legal guardians for participants younger than 18 years old). We excluded participants if the child had advanced cancer with a prognosis of less than 12 months.

The comparison group was recruited retrospectively from patients treated at the same institution between 2013 and 2015 who received standard care and had not participated in the *VIE project*. This approach allowed a preliminary comparison of outcomes between participants exposed to the HP program and those who received standard care alone.

Ethical approval for this study was obtained from the CHUSJ Research Ethics Board and the study was conducted in accordance with the Declaration of Helsinki. Written informed consent was obtained from parents or adolescents ≥18 years old and verbal consent for children and adolescents <18 years old.

### 2.4 Procedure and data collection

The study consisted of self-report questionnaires with parents, semi-structured interviews with adolescents and parents of children affected by cancer, and functional assessments with children and adolescents affected by cancer.

Acceptability questionnaires were sent by email to all families who participated in the *VIE project* by the study coordinator as part of the end-of-program survey regrouping questionnaires from all the study components. Families completed the questionnaire independently at a convenient time and returned it electronically. Exit interviews and functional assessments were conducted during the end-of-program assessments. Participants were contacted by telephone by the study coordinator to schedule the assessments during a visit at the oncology clinic. Exit interviews were conducted with a subgroup of participants to assess acceptability in more depth as well as perceived benefits of having participated in the *VIE project*. A convenience sample of adolescents and parents who were available and had not participated in a previous interview during the study were invited to participate in the interview. When both adolescent and parent from the same family agreed to participate, interviews were conducted jointly for convenience unless participants preferred to be interviewed separately. Interviews were audio-recorded, transcribed verbatim, and anonymized. The interviewer (C.D.) had prior clinical and research experience in pediatric oncology rehabilitation, which facilitated rapport with participants while also requiring reflexive consideration of potential assumptions during data interpretation. Reflexive discussions were conducted during analysis to consider how prior experience in pediatric oncology rehabilitation could influence interpretation of findings.

### 2.5 Measures

#### 2.5.1 Acceptability

We collected qualitative and quantitative data on acceptability of the program using a questionnaire and semi-structured interviews.

The program's acceptability questionnaire was measured using a 7-item questionnaire for parents specifically developed for this study. Three questions were adapted from an existing acceptability questionnaire, the acceptability of treatment programmes questionnaire from the Specialist Parkinson's Integrated Rehabilitation Team Trial (31), for which psychometric properties were not reported. Four additional questions were added following a discussion with other members of the research team based on the research questions. The final version of the questionnaire was revised by experts in the field and a Resource Patient. The questionnaire consisted of seven questions rated on a 5-point Likert scale (0 = strongly disagree—very unsatisfied to 4 = strongly agree—very satisfied) that related to the different aspects of the program (e.g., team, support received, complementarity of the interventions) and general satisfaction. It also included an open box to collect any additional comment.

Exit interviews followed a semi-structured interview guide, which was adjusted as new information emerged. Major themes addressed were (1) general experience in the program, (2) perceived benefits of the program, and (3) recommendations for potential improvement. The order and the wording of questions were adapted flexibly to facilitate an open and natural conversation, and the formulations were adapted according to the age of the participant.

#### 2.5.2 Functional outcome

Assessments to evaluate functional outcomes were chosen collaboratively with the rehabilitation professionals based on feasibility and their clinical relevance. All assessments were performed by a trained occupational therapist and/or physiotherapist. The AMPS was used for children and adolescents aged ≥10 years old (32). It is a standardized objective measure of the quality of activities of daily living (ADL) task performance. It evaluates 16 motor skills and 20 process skills that are the smallest observable units of ADL task performance, and the scores are transformed by age-related norms into a percentile score. AMPS skills are goal-directed actions, and the quality of each skill is evaluated within the context of the person performing daily life tasks. The AMPS is reported as the best measure to evaluate ADL performance or capacity for children and adolescents of all ages and it is the only measure of ADL that evaluates underlying motor and cognitive deficits in task performance (33). Furthermore, children typically enjoy and engage willingly in this assessment (34), and it has been used previously in this population (35–38). However, considering that the assessments took place at the hospital, it was not feasible to replicate a home environment for younger children to perform simple ADL such as eating a meal and getting dressed like they would at home. Thus, the Movement Assessment Battery for Children, second edition (MABC-2) was used for children <10 years old. The MABC-2 (39) is an individually administered standardized measure of movement impairment for children three to 16 years of age that consists of eight standardized tasks divided in three subsections: hand function, ball skills, and balance skills. This test has fair to good reliability (39–41), excellent content validity (39, 42), and has been previously used in this population (43–48). The total score of the MABC-2 is transformed by age-related norms into a percentile score. The AMPS and MABC-2 were selected to allow assessment of functional performance across a wide developmental range. Both assessments produce age-normed percentile scores that permit comparison relative to peers.

### 2.6 Sample size

We calculated the sample size required for the functional outcome, for which a multiple linear regression test was required based on a dichotomous predictor variable and a continuous outcome with confounders. Green (49) indicates that adequate power (80%) can be achieved for moderate effect sizes with a sample size *n* > 50 + 8 m, where *m* is the number of covariates to be modelled. There were no covariates in this study. We adjusted for four potential confounders (i.e., age, sex, type of cancer and time since end of treatment) by adding 1 additional subject per level (1) or per degree of freedom (df), thus a total of 58 participants were required.

### 2.7 Data analysis

Participants’ characteristics were described using medians, ranges, and percentages. Normality assumptions of continuous variable were verified using the Shapiro–Wilk test. The distributions of participants’ characteristics were compared using Mann–Whitney *U* test for continuous variables and Chi square-test for categorical variable, between (a) intervention group and control group, (b) participants and non-participants in the intervention group, and (c) participants and non-participants in the control group.

For the functional outcome data, we first checked for assumption of normality, homoscedasticity, and absence of multicollinearity. As the normality assumption was violated, we used a log-transformation as a correction strategy. Significance of the difference in the mean for motor and cognitive abilities between both groups were determined using multiple linear regression (*p* < 0.05), adjusting for age, sex, type of cancer, and time since end of treatment as confounders. For motor abilities, results for the MABC-2 were used for the participants <10 years old or the AMPS for participants ≥10 years old, as they are scored on the same scale (i.e., percentiles). For the cognitive abilities, only the percentile score from the AMPS assessment were used for the participants ≥10 years old, as this measure was not available for younger participants. In addition, frequencies and percentages of scores under the test norms were reported. Scoring resulted in two descriptive categories: normal (>pc15, >−1SD), or below average scores (pc ≤ 15, ≤−1SD). Statistical analyses were performed using SPSS (Version 28).

For qualitative data, thematic analysis was used with NVivo Software (Version 13). Interview transcripts were analyzed inductively using an iterative thematic analysis approach (50), as no theoretical background or prior evidence was available to guide the development of themes. The first author (C.D.) conducted the first coding, with ongoing discussions with a second researcher (J.K.) to refine code definitions, challenge interpretations, and consolidate emerging themes. Coding and theme development occurred concurrently and iteratively across transcripts, allowing new insights to inform subsequent analyses. This process continued until consensus was reached that the final themes adequately captured patterns across the dataset while also transcending individual interviews.

Subsequently, to integrate the quantitative and qualitative findings obtained separately, we integrated the findings using a side-by-side table and discussed how the qualitative results either confirm, disconfirm, or complement the quantitative results.

## 3 Results

### 3.1 Sample and clinical characteristics

**Figure 1:**
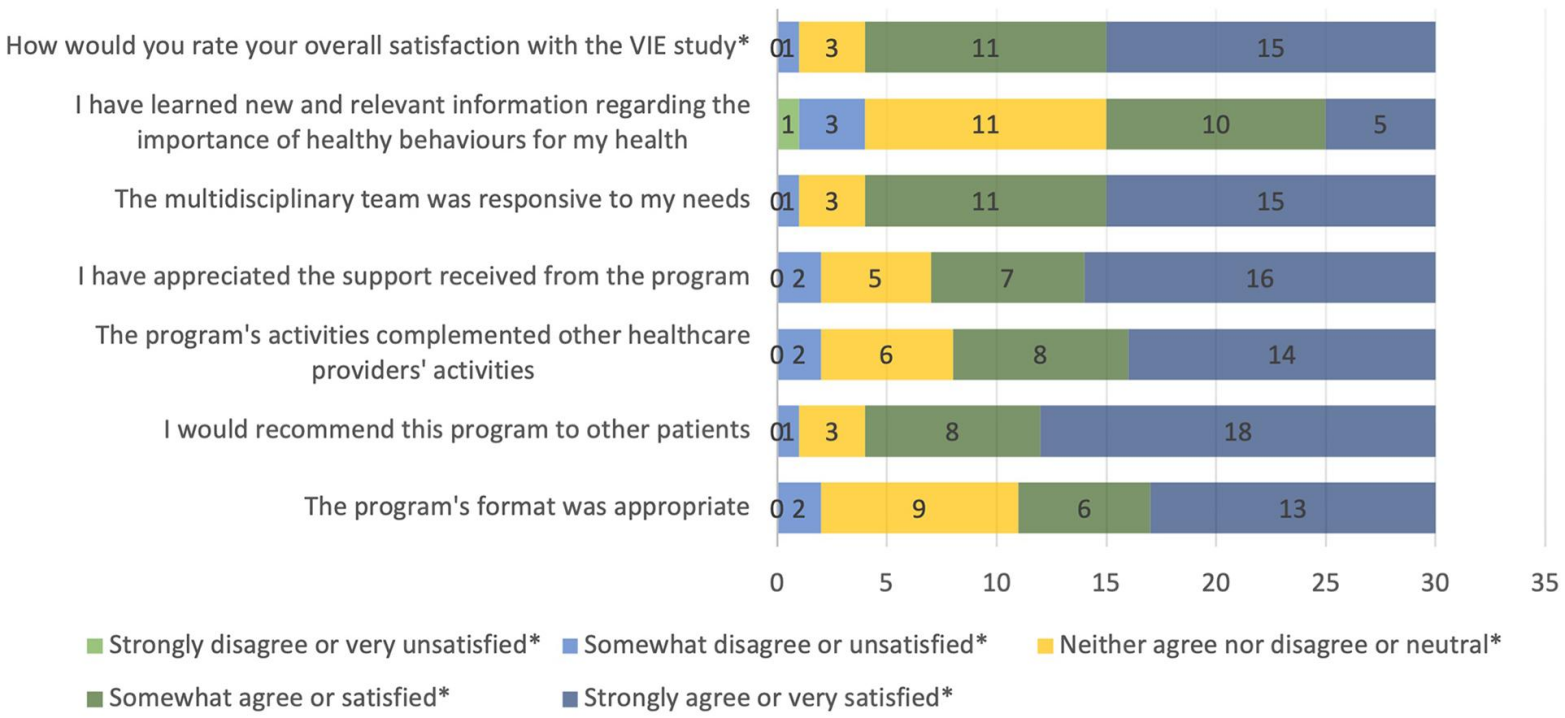
Flow chart of participants in the intervention group.

For the intervention group, 62 participants were recruited over the 2-year recruitment period, with a recruitment rate of 67%. Five participants died and four dropped-out of the study, resulting in 53 participants eligible for the end-of-program assessment and 45 assessed (see Figure 1). Eighty-two participants were recruited for the control group. For feasibility reasons (i.e., non-availability of the facilities or therapist for assessment), it was not possible to conduct the functional assessment with all the participants from the control group, and a convenient sample of 29 participants was recruited (i.e., 35.4% recruitment rate). Consequently, the study was adequately powered for analyses of motor abilities but underpowered for cognitive abilities analyses.

Among the 74 participants (45 in intervention group, 29 in control group), approximately half were male (48.6%) and ages ranged from 3 to 18 years (median 7). Time since diagnosis ranged from 18 to 48 months (median 28) and time since end of treatment from 0 to 31 months (median 12), with 10 participants still receiving treatments or who had completed treatment less than 1 month. About half of participants (*n* = 36, 48.6%) had a diagnosis of leukemia, 12 (16.2%) of lymphoma, six (8.1%) of central nervous system tumour, and 18 (27.0%) had other diagnoses. When comparing sociodemographic and clinical characteristics of the different groups (i.e., intervention group vs. control group, participants vs. non-participants in intervention group, participants vs. non-participants in control group) in relation to sociodemographic and clinical characteristics, we found that time since end of treatment was longer in the intervention group, compared to the control group (*U* = 421, *p* < 0.05), and that age at diagnosis was lower for participants in the control group, compared to non-participants. No other statistical differences were found (Table 2). Participation in each program component can be found in Table 3. The number of sessions received varied considerably between participants and across program components, particularly for the PA and psychosocial interventions. Some participants received no intervention in specific components, whereas others received substantially higher levels of exposure. This variability reflects the individualized and flexible nature of the program, which was designed to adapt to participants’ needs and treatment trajectory.

**Table 2:** Characteristics of the study participants (*n* = 74).

Characteristics	Intervention group (*n* = 45) *n* (%) or median (range)	Control group (*n* = 29) *n* (%) or median (range)	Whitman-*U* or *X*^2^ intervention vs. control group	*p* intervention vs. control group	Non-participants intervention group (*n* = 17) *n* (%) or median (range)	Non-participants control group (*n* = 53) *n* (%) or median (range)
Sex						
Male	24 (53.3)	12 (41.4)	1.01	0.32	8 (47.1)	24 (45.2)
Female	21 (46.7)	17 (58.6)			9 (52.9)	29 (54.7)
Type of cancer						
Leukemia	23 (51.1)	13 (44.8)	0.28	0.60	7 (41.2)	24 (45.3)
Other	22 (48.9)	16 (55.2)			10 (58.8)	29 (54.7)
Age at diagnosis (years)	8 (3–18)	7 (4–18)	626	0.76	13 (1–17)	10 (5–21)*
Time since diagnosis (months)	28 (20–48)	28 (18–47)	618	0.70	N/A	N/A
Time since end of treatment (months)	16 (0–31)	10 (0–23)	421	0.01*	N/A	N/A

* *p* value < 0.05 between participants intervention group vs. non-participants intervention group and between participants control group vs. non-participants control group.

**Table 3:** Exposure to components of the program.

Program component	Mean (SD)	Median	Minimum	Maximum	Interquartile range
Psychosocial component	1.86 (2.69)	1.00	0	12	3
Physical activity component	17.32 (16.31)	11.00	0	73	14
Nutrition component	4.55 (1.59)	5.00	1	7	2

### 3.2 Acceptability

**Figure 2:**
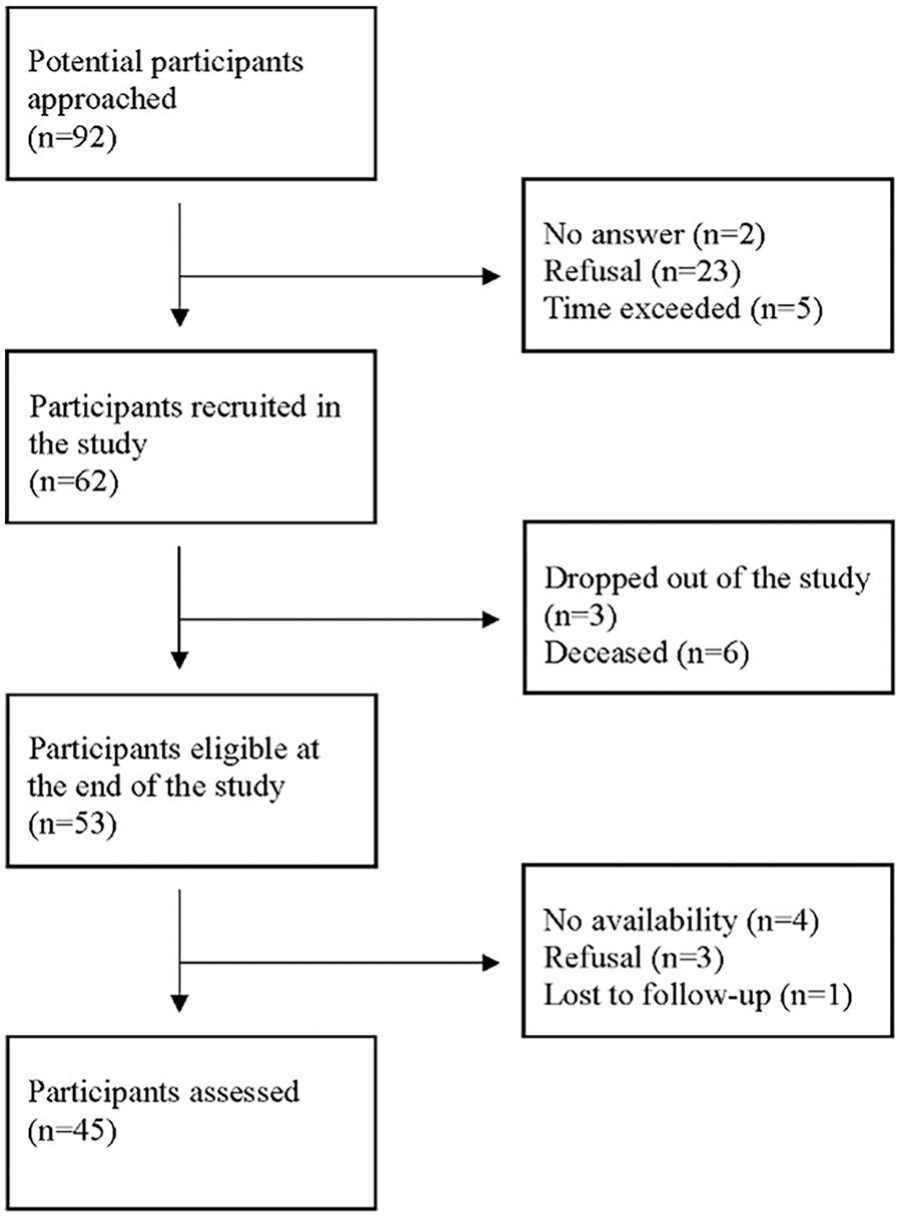
Acceptability questionnaire.

The acceptability questionnaire was completed by 30 (56.6%) participants. Figure 2 shows the responses obtained. Nearly nine out of ten participants ten reported that they would recommend the program to other families (86.7%) and indicated they were either satisfied or very satisfied with the current program (86.7%). In addition, the majority of participants found that the multidisciplinary team was responsive to their needs, that the interventions were complementary with other clinical activities, and that the program format was suitable (86.7%, 73.3%, and 63.3% somewhat agree or strongly agree, respectively). They also appreciated the support provided by the program (76.7% somewhat agree or strongly agree). Half of the participants agreed that they learned new and useful information regarding the importance of adopting healthy behaviours (50.0% somewhat agree or strongly agree).

All the exit interviews were conducted by the first author (C.D.) between May 2021 and March 2022, face-to-face (*n* = 13) or virtually (*n* = 1). Fourteen families were approached, all of which accepted. As four families had two participants (i.e., one adolescent and one parent), 18 individuals were interviewed. After interviewing 18 participants, data saturation was considered reached. Twelve participants were parents (eight mothers, four fathers) and six were adolescents (range 12–17 years old) (see Table 4). The interviews ranged in duration between 5 and 26 min (median 12 min). The results are presented according to the overarching themes covered in the interviews.

**Table 4:** Characteristics of interviewed participants (*n* = 18).

Characteristics	Adolescents (*n* = 6) median (range) or *n* (%)	Parents (*n* = 12) median (range) or *n* (%)
Age of the child or adolescents (years)	13.5 (13–17)	10.5 (5–17)
Sex		
Male	3 (50.0)	2 (17)
Female	3 (50.0)	10 (83)
Race/ethnicity		
White/Caucasian	6 (100.0)	12 (100.0)
Other	0 (0.0)	0 (0.0)
Type of cancer		
Leukemia	4 (60.0)	5 (41.7)
Lymphoma	1 (20.0)	2 (16.7)
Central nervous system tumors	0 (0.0)	2 (16.7)
Extra cranial solid tumor	1 (20.0)	3 (25.0)
Education level		
Unfinished high school	N/A	2 (16.7)
High school or professional school	N/A	6 (50.0)
University, bachelor degree	N/A	4 (33.3)
University, graduate degree	N/A	0 (0)

#### 3.2.1 Theme 1: Experience in the program

Overall, participants reported having a positive experience in the program: “*It was like a silver lining with everything that had happened… It allowed [my son] to have fun, through it all. When he was coming to the hospital, it was fun to do [PA interventions]. Or, by Zoom, it was a privilege for him. He could benefit from that, when nobody else could*” (Mother of a 13-year-old participant). The positive aspects of the program identified were the adaptability and flexibility in the delivery of interventions, and the trust and positive relationships established with the intervention deliverers. Participants appeared to value relational and organizational aspects of program delivery more than the intervention content itself. Flexibility, personalization, and continuity of support emerged as central mechanisms underlying acceptability. Frequently reported negative aspects of the program included completing numerous lengthy questionnaires (e.g., three-day food records) and the burden of the cancer experience that prevented them from participating: “*At a certain point in time, […] particularly at the end, it's too much of everything. […] I think at a certain point, we are like overwhelmed”* (Mother of a 7-year-old participant). Regarding the experience with remote interventions, some participants reported that it resulted in increased participation while others were not interested.

#### 3.2.2 Theme 2: Perceived benefits of the program

The main perceived benefit of participating in the program was the improvement in their patient experience. The findings suggest that perceived value of the program may not have been primarily linked to measurable functional improvement, but rather to emotional support, normalization of the hospital experience, and opportunities for meaningful engagement during treatment. For example, the PA component in particular allowed children to have fun, get distracted from the treatment, and be busy during the long days at the hospital. Another important perceived benefit was access to counselling and additional support aimed at improving health behaviours: “*If I had not seen the kinesiologists, I would probably not have had… well, had been willing to go back to practicing sport. And I would still be depressed. So, I would have just gotten worst. So, it really helped me”* (12-year-old participant).

Regarding the direct impact on the adverse effects of cancer or their functional outcome, some participants reported positive changes in their health, resulting from the participation in the program: “*It was giving more energy. So I was less tired. It was easier to do my activities”* (13-year-old participant). One participant reported suffering less from fatigue and thus being able to go back to his daily activities faster than expected (e.g., attending the regular physical education courses at school). However, most participants did not perceive a direct or indirect impact of the program on their health or functional outcome.

#### 3.2.3 Theme 3: Suggestions for improvement

Suggestions for program improvements were provided for each program component. For the PA component, it was suggested to have more material (e.g., stationary bikes) and more availability (i.e., every day of the week). For the nutrition component, participants would have appreciated having group or individual kitchen activities. For the psychosocial component, more flexibility in the scheduling would have increased participation rate as it was offered near diagnosis, at a moment when many parents felt too overwhelmed, tired, or preoccupied to engage in the proposed intervention.

### 3.3 Functional outcome

Regarding feasibility, the functional assessments were conducted with 84.9% of eligible participants from the intervention group. Reasons for non-evaluation included no availability (*n* = 4), refusal (*n* = 3), or lost to follow-up (*n* = 1). The multiple regression analysis revealed no significant difference between the intervention and control groups for motor (*p* > 0.05) and cognitive abilities (*p* > 0.05), after adjusting for age, sex, type of cancer, and time since end of treatment. All 75 participants assessed had results for motor abilities and 31 participants ≥10 years old also had results for cognitive abilities. In both groups (i.e., intervention and control groups), 25 (33.8%) participants had motor abilities under the age norms (41.9% for MABC-2 and 22.6% for the AMPS) and two (6.7%) for cognitive abilities. In the control group, 7 (24.1%) had motor abilities under the age norms (i.e., ≤15th percentile) and one (7.1%) for cognitive abilities.

### 3.4 Integration

Table 5 presents a joint display of quantitative and qualitative findings. Integration of findings revealed a divergence between participants’ perceived value of the program and objectively measured functional outcomes. Although quantitative analyses did not demonstrate statistically significant improvements in functional outcomes, qualitative findings suggested that participants perceived substantial psychosocial and experiential benefits. Families frequently described the program as supportive, motivating, and emotionally meaningful during treatment, indicating that intervention acceptability may operate independently from measurable functional change.

**Table 5:** Joint display of results.

Quantitative results	Quotes
Acceptability and view of the program	
50.0% of participants reported that they did not learn new and relevant information (neither agree nor disagree; somewhat disagree; or strongly disagree)	“It was very nice, but what I would have liked, would be to have had more explanation” (Mother of a 5-year-old participant)
86.7% of participants reported that they would recommend the program to other patients (somewhat agree or strongly agree) and 86.7% that they were satisfied (satisfied or very satisfied)	“Honestly, I think that a lot of children could benefit from that. Especially during a pandemic when you can't get out of your room. It was important” (Mother of a 7-year-old participant)
	“I hope that it continues. […] I wish it for the children, I wish it very much because, because it helps” (Mother of a 7-year-old).
76.7% of participants appreciated the support provided by the program (somewhat agree or strongly agree)	“We feel supported, through the years, the 2 year of treatment. You know, when we have questions, they can counsel us a little.” (Mother of a 13-year-old participant)
	“Personally, I want to say a big thank you to the team that has shown adaptation skills and remarkable flexibility, and that, we have appreciated it a lot” (Father of a 13-year-old participant)
Functional outcome and view of the program	
No significant difference for motor abilities, between intervention and control group (*p* > 0.05)	“I cannot say there has been a major impact. Of course it is always good to have alternatives on the side you can take, especially when you are going through an illness” (Father of a 8-year-old participant)
	“Not really improved my [fitness] because I haven't done enough [PA interventions] to really be able to say something about that” (17-year-old participant)
No significant difference for cognitive abilities, between intervention and control group (*p* > 0.05)	“The doctor tells us, that some can develo*p* … because they take so much medications, attention deficits disorders or something else, so maybe more exercises regarding concentration, it would have been, well in my case, I would have appreciated it” (Mother of a 5-year-old participant)

The qualitative findings also provided important contextual insight into the quantitative results, helping explain why participants reported high acceptability despite limited measurable functional benefits. Indeed, all participants with very low functional outcome (i.e., ≤5th percentile) who completed the acceptability survey reported being satisfied or very satisfied with the program. Similarly, average functional percentiles were comparable between participants reporting low satisfaction (i.e., neutral or unsatisfied) and those reporting high satisfaction (i.e., satisfied or very satisfied), both for motor outcomes (25.8 vs. 30.2 percentile, respectively) and process outcomes (25.0 vs. 25.9 percentile, respectively). Together, these findings suggest that participants valued the program for reasons extending beyond measurable functional improvement.

## 4 Discussion

### 4.1 Summary of main findings

This study aimed to assess the acceptability of a HP program, explore the views of participants regarding their experience, and to examine the program's preliminary effectiveness in improving the functional outcome of children or adolescents affected by cancer. Overall, findings suggest that the *VIE project* is perceived as acceptable by families who participated in the evaluation and that it contributed positively to the patient and family experience during their cancer journey. However, we found no functional benefit for the group who received HP interventions in addition to standard care over standard care only. The qualitative findings provide important context for interpreting these results, indicating that while families valued the support provided by the program, many participants did not perceive clear functional benefits from their participation 2 years after diagnosis.

In our cohort, 41.9% of participants <10 years old assessed using the MABC-2 to measure motor performance showed impaired motor outcome, which is slightly higher than previously reported in other studies that also used this standardised assessment and found that between 25 and 33% of children affected by cancer scored below the 15th percentile (45, 48, 51). For the AMPS assessment, our results are similar to another study (37) that did not report a significant difference between the intervention and control groups after testing the effects of a physically active video gaming on cognitive function and execution of ADL. The authors concluded that their intervention was not intense enough to influence cognitive outcome measures, or that a longer intervention period would have been required to see a measurable effect (37). Similarly, another study evaluating the effect of PA and motivation-based interventions during leukemia treatments concluded that delivering the interventions at the level and intensity required to improve function may not be feasible during early treatment (52).

Several factors may help explain the absence of measurable differences in functional outcomes between groups. First, participation in the program components was individualized and varied across families, which may have limited the intensity or consistency of exposure to the interventions. Second, the timing of certain components within the cancer trajectory may have influenced engagement. As reflected in the qualitative interviews, families often reported feeling overwhelmed during the early phases of treatment, which may have reduced their capacity to fully engage with some program components, particularly those offered shortly after diagnosis. Timing of intervention therefore appears to be a critical factor when designing and implementing programs in this vulnerable population. Finally, the functional outcome measures used in this study may not have been sufficiently sensitive to detect changes related to health behaviour interventions over the study period. Our experience and knowledge of the literature supports the use of outcomes that are as specific as possible to the target of the intervention (53), in this case the change in health behaviours. It is also possible that a longer follow-up would have been needed to see an impact.

Regarding the impact of the program on aspects other than patient experience, helping children and their family to make sense of their experience could help them better understand the short and long-term impact. This could be done by adding education or reflective sessions towards the end of the program. For example, a study evaluating the effect of a psychosocial support program for young adult who were cancer survivors that used a reflective activity on the observed effects of the seminar and its applicability (54).

### 4.2 Strengths and limitations

A major strength of this study is the use of a mixed-method design, which allowed for a more comprehensive understanding of the absence of significant functional improvements findings. Qualitative studies are useful when trying to make sense of why promising clinical interventions do not always work in the real world or how patients experience care (55). Qualitative findings provided important context for interpreting the quantitative results. Interestingly, satisfaction with the program was not related to the functional outcome of children or adolescents. Participants seemed to place more value on the support and positive experience the program offered. Another strength is the use of a functional outcome assessment that evaluates both the physical and cognitive outcomes, as few studies have evaluated the impact of health behaviours on survivors’ cognitive aspects of task performance (56).

This study has several limitations that should be considered when interpreting the results. First, the study included a relatively small and heterogeneous sample, which limited statistical power to detect significant differences in functional outcomes and prevented subgroup analyses (e.g., according to intervention intensity or type of assessment used). The heterogeneity of participants in terms of age, cancer diagnosis, treatment trajectory, and intervention exposure may also have contributed to variability in outcomes and limited the ability to detect significant functional effects at the group level. As such, findings should be interpreted with caution. However, this study was designed as an exploratory investigation focused primarily on feasibility, acceptability, and intervention refinement rather than definitive effectiveness testing. Second, recruitment rates differed substantially between the intervention and control groups, and group allocation was based on treatment status at the time of the study rather than randomization. This non-randomized design may have introduced selection bias and limited comparability between groups. In addition, the cross-sectional comparison at a single time point limited the ability to examine trajectories of functional change over time or establish causal relationships between the intervention and outcomes. The study design also did not permit participant or assessor blinding, which may have influenced outcome reporting and interpretation. Third, the acceptability questionnaire was developed specifically for this study and did not undergo formal psychometric validation. Additionally, the functional outcome measures assessed related but different constructs, with the AMPS focusing on ADL performance and the MABC-2 evaluating motor coordination and movement skills. Combining these measures as indicators of overall motor-related functioning should be interpreted with caution. Finally, participation rates for the functional outcome in the control group and for the acceptability questionnaire were relatively low (35.4% and 56.6%, respectively), which may have introduced additional selection bias. It is possible that families experiencing fewer functional challenges at the end of their cancer treatment were more likely to participate in the control group assessments, while participants with more positive perceptions of the program were more inclined to complete the acceptability evaluation or participate in the interviews. Consequently, the acceptability findings may overrepresent favourable experiences.

The results from this study are supportive of future work that could evaluate the program using a larger randomized design. Future research with a bigger sample size and rigorous methodology, such as a randomized controlled trial, is required to address some of the limitations and measure the impact of the program on children with cancer's functional outcome.

## 5 Conclusion

The HP program was well-accepted and appreciated by families during their cancer journey; however, it was not associated with measurable improvements in functional outcome for the children or adolescents who received the program in addition to standard care. These preliminary findings support the integration of HP programs within pediatric oncology settings and highlight the need for a full-scale trial, incorporating adaptations based on family feedback. Further research is required to enhance the effectiveness of HP interventions in improving functional outcomes for participants.

## Data Availability

The raw data supporting the conclusions of this article will be made available by the authors, without undue reservation.
